# Insulin Resistance, Secretion and Clearance –Taming the Three Effector Encounter of Type 2 Diabetes

**DOI:** 10.3389/fendo.2021.741114

**Published:** 2021-09-29

**Authors:** Jacob Bar-Tana

**Affiliations:** Human Nutrition and Metabolism, Hebrew University Medical School, Jerusalem, Israel

**Keywords:** insulin resistance, insulin secretion, mTORC1 (mechanistic target of rapamycin complex 1), diabetes, metabolic syndrome and type II diabetes

## Introduction

The current paradigm of type 2 pre-diabetes/diabetes (T2D) maintains that glycemic control reflects the interplay between insulin production by beta-cells and the peripheral sensitivity/resistance to insulin. Insulin resistance implies failure of insulin to activate glucose uptake in muscle and adipose tissue and glycogen synthesis in liver and muscle, and to inhibit liver gluconeogenesis and adipose lipolysis. The pre-diabetes stage is considered to present progressive resistance to insulin, being offset by insulin hypersecretion by beta-cells, resulting in maintaining plasma glucose levels within the pre-diabetes limits. The overt diabetes stage that follows presents ‘exhaustion’ of beta cells, resulting in progressive hyperglycemia (1).

The close association between insulin secretion and insulin resistance leaves open the question which comes first (2, 3). Similarly, the association between insulin resistance and hyperinsulinemia in normoglycemic normolipemic off springs of T2D parents (4) is still undefined in terms of primary cause-effect. The classical paradigm maintains that insulin resistance is the primary defect of T2D, followed by ‘compensatory’ increase in beta cells insulin production (1, 5). An alternative paradigm maintains that insulin hypersecretion by beta-cells is the primary defect, resulting in hyperinsulinemia which drives peripheral insulin resistance (6). Each of the two paradigms appears to be substantiated by respective examples (2–6). However, the apparent cause-effect relationship outlined by each still remains unresolved in terms of molecular mode(s)-of-mediation. Thus, in spite of previous attempts (e.g., beta-trophin, irisin) no humoral and/or neuronal agents have yet been identified which may mediate between primary insulin resistance and increase in beta cells mass and function during the early normoglycemic normolipemic pre-diabetes stage of T2D. Also, the mode of suppression of the insulin transduction pathway in liver, muscle and adipose fat by primary hyperinsulinemia still remains unresolved. Moreover, plasma insulin levels are further determined by hepatic insulin clearance, which amounts to >50% of the insulin secreted by beta-cells (7). Indeed, hepatic insulin clearance is significantly inhibited in T2D patients, thereby synergizing with insulin hypersecretion by pre-diabetes beta cells (8, 9). Thus, the egg-chicken riddle of insulin resistance and secretion appears to be further complicated by the three-effector encounter of insulin resistance, secretion and clearance. Solving the concerned three-effector encounter may help in realizing the primary pathological driver and primary target for treatment of T2D.

## The Three-Effector Encounter of T2D Is Driven by mTORC1

**Figure 1:**
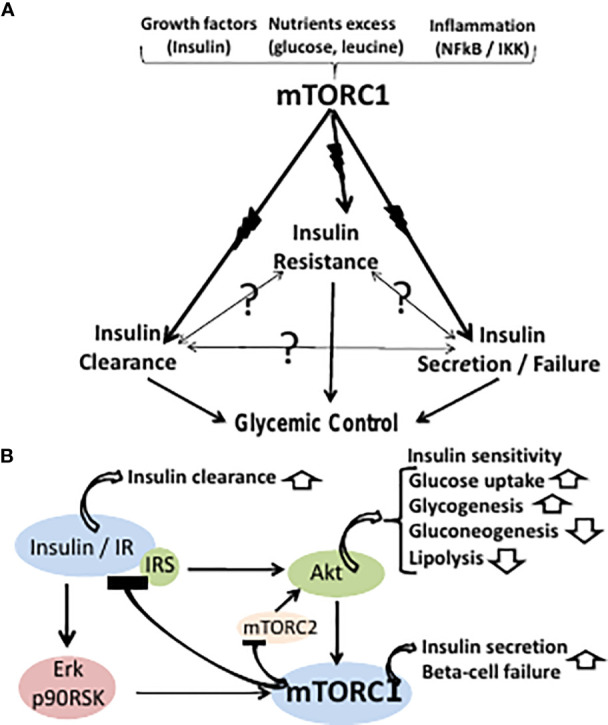
**(A)** The classical paradigm of T2D glycemic control maintains a cause-effect relationship between insulin resistance, secretion and clearance. However, the cause-effect paradigm is still unresolved (symbolized by question marks) in terms of molecular modes-of-mediation. The three effectors of T2D are proposed here to be concomitantly driven by an upstream primary hyperactive mTORC1. mTORC1 may be hyper-activated by growth factors (e.g., insulin), nutrients excess (e.g., glucose, leucine), or inflammation (e.g., NFkB/IKK), while being suppressed by metabolic stress. **(B)** Hyperactive mTORC1 may concomitantly drive peripheral insulin resistance, beta cells insulin hypersecretion and failure, and suppression of hepatic insulin clearance. Thus, hyperactive mTORC1 drives insulin resistance by disrupting the IR-Akt transduction pathway resulting in glycogenolysis, gluconeogenesis, inhibition of glucose uptake and adipose lipolysis. mTORC1 activation by the insulin-IR-Erk transduction pathway allows for sustained activation of mTORC1 by insulin upon disrupting the insulin-IR-Akt transduction pathway. Hyperactivation of beta cells mTORC1 serves as double-edged driver, allowing for insulin hypersecretion, while concomitantly promoting beta cells ER stress and apoptosis. Hepatic hyperactive mTORC1 suppresses hepatic insulin clearance.

In lack of direct cause-effect relations between insulin resistance, secretion and clearance, the three effectors of T2D are proposed to be ***concomitantly*** driven by an upstream primary con-founder, namely, hyper activation of the mammalian target of rapamycin complex 1 (mTORC1) (**Figure 1A**). mTORC1 controls growth and metabolism by phosphorylating and/or affecting its downstream targets S6K1, 4EBP, CRTC2, lipin, ATF4, HIF1a, PPARg, PPARa, ULK1, TFEB, autophagy and others (10). Wildtype mTORC1 kinase activity may be hyper-activated by growth factors (e.g., insulin), energy/nutrients excess (e.g., glucose, leucine, arginine) and inflammation (e.g., NFkB/IKK), while being suppressed by metabolic stress (e.g., caloric, hypoxic, hyperosmotic, redox) (10, 11). Suppression of mTORC1 activity or its downstream targets is reported to ameliorate T2D phenotype in animal models (12, 13) and human (14–16), implying a putative upstream role of hyperactive mTORC1 in driving the three-effector encounter of T2D.

Hyperactive mTORC1 drives insulin resistance by disrupting the insulin receptor (IR)-Akt transduction pathway in liver, muscle and adipose tissue, resulting in glycogenolysis, gluconeogenesis, inhibition of glucose uptake, unrestrained hyperglycemia and adipose lipolysis (17) (**Figure 1B**). Thus, phosphorylation of IRS1,2(Ser312, 636/639) by hyperactive mTORC1 and IRS1,2(Ser307, 1101) by hyperactive S6K1 result in suppressing the phosphorylation of IRS tyrosine(s) followed by IRS ubiquitination and degradation (18, 19). Also, phosphorylation and stabilization of GRB10 by hyperactive mTORC1 results in disrupting the IR/IRS transducer (20, 21). The IR-Akt transduction pathway is further disrupted by suppression of Akt(Ser473) phosphorylation by mTORC2, due to inhibition of mTORC2 kinase activity by hyperactive mTORC1/S6K1 (22, 23). Of note, disruption of the IR-Akt transduction pathway by hyperactive mTORC1 may still allow for sustained activation of mTORC1 by insulin, being mediated by the IR-Erk1,2 transduction pathway, implying a functional redundancy of the Akt and the Erk1,2 effectors in mediating mTORC1 activation by insulin (24) (**Figure 1B**).

mTORC1 drives beta-cells proliferation, cell size and insulin production (25). Hence, concomitant hyper activation of mTORC1 in liver, muscle, adipose fat and beta-cells may account for the close association between peripheral insulin resistance and insulin hypersecretion during the pre-diabetes stage of T2D (**Figure 1B**). However, hyper activation of beta cells mTORC1 serves as double-edged driver, allowing for beta cells high performance, while concomitantly promoting beta cells ER stress and apoptosis (26–28). Thus, chronic increased production of insulin and its islet amyloid polypeptide (IAPP) by-product may result in unfolded protein response (UPR), aimed at eliminating surplus by suppressing protein synthesis while increasing lysosomal autophagy and/or proteasome degradation. However, these degradation pathways are blocked by hyperactive mTORC1, resulting in apoptosis due to unresolved ER stress (29). Also, disruption of beta cells IR-Akt-FOXO1 pathway by hyperactive mTORC1 results in suppressing PDX1 and beta cells survival (30), while promoting alpha-cells glucagon expression (31) and glucagon-induced hepatic gluconeogenesis. The two concomitant contrasting aspects of beta-cells hyperactive mTORC1 may dynamically evolve during the clinical sequel of T2D, whereby the hyperplastic-hypertrophic pre-diabetes phase yields progressively to overt T2D beta-cells failure (1). Indeed, the decline in beta cells disposition index starts within the range of normal glucose tolerance and progressively deteriorates as patients progress to hyperinsulinemic prediabetes and then to overt T2D (1). Hence, the current two-stage paradigm of pre-diabetes/diabetes overlooks the inherent pathophysiological continuum of T2D.

Hepatic hyperactive mTORC1 suppresses hepatic insulin clearance, which depends on IR availability, and further depends on CEACAM1 phosphorylation by functional IR. Thus, hepatic insulin clearance is mediated by insulin binding to the IR followed by endocytosis, lysosomal insulin degradation and IR recycling to the cell membrane (32). Hyperactive mTORC1 phosphorylates and stabilizes GRB10(Ser476), resulting in its binding to IR, IR ubiquitination and degradation (33) (**Figure 1B**).

Hyperactive mTORC1 may further revoke a primary role of ‘lipotoxicity’ in driving the three effectors of T2D (34). Indeed, downstream mTORC1 targets [e.g., SREBP, CRTC2, Lipin and PPARalpha (10)] may drive the increase in long-chain fatty acyl-CoAs and the accumulation of diglycerides and ceramides in non-adipose tissues. Secondary ‘glucolipotoxicity’ may complement hyper-active mTORC1 in shaping the phenotype of overt T2D.

## Discussion

Concomitant driving of the three-effectors of T2D by hyperactive mTORC1 turns redundant the question which effector comes first. However, mTORC1 activity is affected by genetic, epigenetic, ethnic and/or tissue-dependent factors which may determine its sensitivity to environmental and metabolic conditions. Hence, modulation of each of the three concerned effectors by hyperactive mTORC1 may be context-dependent, allowing for one effector to precede the others, thereby displaying an apparent cause-effect relationship between the three effectors (2–6).

Most importantly, the primary role played by hyperactive mTORC1 in driving the three-effector encounter of T2D implies that suppression of hyperactive mTORC1 may offer an all-in-one treatment for T2D. That is in contrast to targeting the concerned effectors individually by means of insulin sensitizers, potassium channel modulators, incretins, SGLT2 inhibitors, insulin degrading enzymes and other (35). Hyperactive mTORC1 may indeed be targeted by caloric/carbohydrate restriction (36, 37) and physical exercise (38). However, the compliance to behavioral modification is poor. Also, treatment of T2D patients with rapalogs is dubious since chronic treatment may result in inhibition of mTORC2, thereby suppressing the IR-Akt transduction pathway (39). By-passing that difficulty by intermittent rapalogs treatment (40) still remains to be verified under real life conditions.

Alternatively, hyperactive mTORC1 may be tamed by mitochondrial complex I inhibitors. Suppression of hyperactive mTORC1 due to inhibition of mitochondrial complex I is best exemplified by metformin (41–43) used as first-line therapy for T2D (44). The anti-diabetic efficacy of pioglitazone may similarly be ascribed to suppression of mTORC1 kinase activity due to inhibition of mitochondrial complex I (45, 46). Also, classical mitochondrial complex I inhibitors (e.g., rotenone) are reported to alleviate insulin resistance and beta-cell failure in T2D animal models (47). Suppression of hyperactive mTORC1 by mitochondrial complex I inhibitors may be ascribed to redox (NADH/NAD), energy (ATP/AMP), AMPK and/or oxidative (ROS) stress as function of the respective dose (11, 43). Since mTORC1 controls disease aspects of T2D beyond glycemic control (10, 17), suppression of mTORC1 kinase activity by metformin may further account for its pleiotropic effects in improving health- and life-span (48). The mitochondrial/mTORC1 connection may prompt a search for novel mitochondrial complex I inhibitors (49) which may tame hyperactive mTORC1 and the three-effector encounter of T2D.

## Author Contributions

Composed and submitted by JB-T. The author confirms being the sole contributor of this work and has approved it for publication.

## Conflict of Interest

The author declares that the research was conducted in the absence of any commercial or financial relationships that could be construed as a potential conflict of interest.

## Publisher’s Note

All claims expressed in this article are solely those of the authors and do not necessarily represent those of their affiliated organizations, or those of the publisher, the editors and the reviewers. Any product that may be evaluated in this article, or claim that may be made by its manufacturer, is not guaranteed or endorsed by the publisher.
